# From pathogenesis to therapy: extracellular vesicles in osteoarthritis

**DOI:** 10.3389/fbioe.2026.1882483

**Published:** 2026-08-06

**Authors:** Tarlan Eslami Arshaghi, Aline M. Morrison, Mary Murphy, Frank Barry

**Affiliations:** Institute for Clinical Trials, University of Galway, Galway, Ireland

**Keywords:** disease modification, extracellular vesicle, mesenchymal stromal cell, osteoarthritis, regenerative medicine

## Abstract

Extracellular vesicles are membrane-bound nanoparticles that act as important elements in cell-cell signaling. Consisting of a cargo of proteins, lipids and nucleic acids, they carry information between secreting cells and target cells and induce a phenotypic or functional change in the latter. Thus, they play a multiplicity of roles in modulating physiological responses in healthy tissues. They are also active in a number of pathological conditions where they have an impact on disease progression. In recent years, EVs from several cell sources have been assessed to determine their utility as therapeutic agents to elicit an effective repair or regenerative response in diseased or injured tissues. In this regard, EVs are considered as a promising new biomedicine with the potential to overcome some of the challenges associated with conventional cell therapy. In the diarthrodial joint, EVs are produced by many cells, including chondrocytes, synovial fibroblasts, macrophages and leukocytes, where they play important roles in maintaining tissue homeostasis. They are also involved in the progression of osteoarthritis and other joint conditions. Recent compelling data also indicate that EVs transplanted to the OA joint may act as effective disease modifiers. This review provides an assessment of the functions of EVs in maintaining joint health by virtue of their multifaceted signaling activities. Their role as agents of OA progression is also discussed, as well as their therapeutic potential.

## 1 Introduction

Osteoarthritis (OA) is one of the most common musculoskeletal diseases affecting millions of people worldwide of different age and gender. A combination of trauma, risk factors, biochemical and biomechanical changes, and aging results in an imbalance of articular cartilage and subchondral bone homeostasis. Changes to articular cartilage bone remodeling and sclerosis then leads to ligament and muscle weakness. Disease progression frequently results in painful and swollen joints with limited range of motion. OA has no known cure, with current treatments primarily targeted to symptom management rather than control of disease development. Exercise, weight management, physical therapy, pain medications and ultimately joint replacement remain the available treatments according to the Osteoarthritis Research Society International (OARSI) (Zhang et al., 2008). OA is considered a disease of the whole joint and due to its complex and multifaceted nature and the lack of appropriate treatments, advanced and novel therapies with the intention of restoring structure and function. Advanced therapies include cell-based treatments, bone remodeling modulators, platelet-rich plasma (PRP), cytokine inhibitors, and gene therapy (Roubille et al., 2013). In terms of cell-based therapies, mesenchymal stem cells (MSCs) have been at the forefront of efforts to identify alternative approaches for treatment of OA. However, recent clinical trial data failed in supporting the therapeutic effectiveness of MSCs (Mautner et al., 2023; Pers et al., 2025); yet more research is required. Extracellular vesicles (EVs) secreted by MSCs have been identified as a key element mediating the multidimensional effects of these cells. As a result, the use of EVs alone as a therapeutic intervention has been proposed as a new, safe and effective strategy. This has given rise to the possibility of replacing MSC therapy with MSC-derived EV therapy as a cell-free and off-the-shelf medication. In this review, we explore the role of EVs in OA development and treatment, highlighting the limitations of MSCs and capacity of their EVs to overcome these limitations to answer a key question: “Do EVs have superior potential compared to MSCs to overcome MSC limitations?”

Beyond the limitations described above, MSCs have biological risks that cause the field to shift towards finding an alternative approach of using their EVs to provide safety and efficacy at the same time. First, immunogenicity of MSCs is still one of the main concerns after injections, in particular for allogeneic sources: although MSCs are considered immune-privileged, allogeneic MSCs express MHC class I molecules and can upregulate MHC class II and co-stimulatory molecules under inflammatory conditions, potentially triggering host immune responses and limiting their persistence and efficacy *in vivo* (Ankrum et al., 2014). Second, tumorigenicity is a safety concern associated with some cell products; long-term culture of MSCs can lead to genomic instability, and their pro-angiogenic and immunomodulatory properties raise concerns about possible tumour growth or progression in vulnerable patients (Barkholt et al., 2013). Third, the risk of unwanted *in vivo* behavior of MSCs such as ectopic differentiation represents an additional safety consideration. EVs offer several key advantages that directly address these concerns. They lack nucleus or genomic content that make them non-replicating and therefore without a capacity for ectopic differentiation or uncontrolled proliferation causing tumour formation (Gowen et al., 2020). They express lower levels of MHC molecules and do not present the same surface co-stimulatory signals that trigger adaptive immune responses, making them more suitable for allogeneic, off-the-shelf administration. Taken together, EVs are a promising cell-free alternative that may represent paracrine therapeutic effects of MSCs while overcoming several safety and translational barriers associated with MSC therapy.

## 2 Current MSC position in osteoarthritis treatments and their clinical limitations

MSCs initially gained considerable attention as a regenerative therapy for OA because of their biological properties and unique therapeutic potential. Multi-linage differentiation capacity of the MSCs alongside their immunosuppressive functions made them an attractive candidate for cartilage repair, modulating the inflammatory environment of OA joint, and other degenerative conditions. While their features and their safety profile at the pre-clinical level led them to enter the clinical world, multiple large-scale clinical trials have failed in demonstrating their effectiveness. From over 1,600 clinical trials registered on ClinicalTrials.gov for MSCs, only a small portion reached phase III and mostly remained at early phases (I, II, or I/II). Also, a majority of these trials have no publicly available results. For example, a systematic review on the MSCs clinical trials on musculoskeletal disorders demonstrated that 52 out of 124 trials had neither posted or published results suggesting the possibility of lack of transparency in negative or inconclusive trial outcomes (Holtedahl and Brox, 2024). Despite the sufficient evidence of MSC safety at the clinical scale, many trials failed in demonstrating their efficacy under distinct conditions.

What explains the contradictory results from efficient pre-clinical studies to failed trials? The first and major difference between larger clinical trials and *in vitro* experiments is the controlled environment in smaller scales, where only one or two variables affect the outcomes of the study; in larger clinical trials, multiple factors can alter the desirable outcome.

### 2.1 MSC product variability in quality and manufacturing

Tissue sources, donor characterization and culture methods can affect the quality and efficacy of the MSCs. Lack of critical quality control (QC) assessments before administration and standardisedassays to measure the effectiveness and immunomodulatory capacity of each dose can be considered as one of the reasons underpinning inconsistent therapeutic effects. QC assessments should be disease-specific and designed to ensure their effects in each condition. Moreover, assessing the effects of cryopreservation as compared to fresh samples needs to be understood. This has been demonstrated clearly by the study conducted by Stroncek *et al* (Stroncek et al., 2020); these authors aimed to highlight the multi-lab environmental effects on the same bone marrow samples. This study indicated that cells from the same donors across laboratories demonstrated variability ranging between 23% and 96%, with significant variability in immunosuppressive properties seen. MSCs cultured in Fetal Bovine Serum (FBS) showed higher proliferation and lower immunomodulation in comparison to human Platelet Lysate (hPL) supplemented MSCs. While it has been shown that variability in manufacturing contributes more to the inconsistency than donors, donor-to-donor variations have impacted clinical trial data. Donor age has not been reported as an element contributing to clinical trial failures and there is no clinical trial on MSCs that attribute their negative results primarily to the age of the donors. However, pre-clinical data has demonstrated the limited proliferation and differentiation capacity of MSCs from elderly donors (Heyman et al., 2025; Carvalho et al., 2021).

### 2.2 Heterogenicity in studied populations

Baseline disease severity, inflammation status and patient classification also affected the outcome of clinical trials and their failure, at least in part. For example, in the POSEIDON-DCM phase II trial investigating the therapeutic role of MSCs in non-ischemic dilated cardiomyopathy (DCM), variable efficacy outcomes were demonstrated to be associated with disease heterogenicity in heart failure and inflammation severity. Patients with more severe conditions responded less consistently to MSC injection. In addition, the Prochymal (Remestemcel-L) phase III Trial outcome was affected by the variability of patient inflammatory status and disease stage at their enrollment. While subgroups of patients showed disease improvement, the large and diverse trial cohort led to failure in meeting the primary endpoint in the overall studied population.

### 2.3 Trial design and conduct

There is no consistent and standardisedprotocol for designing and conducting clinical trials. Different MSC dose, timing of administration, and follow-up periods during the trial design can be considered as contributor to the outcome variability and failure of MSCs trials.

### 2.4 Regulatory and reporting issues

As stated above, there is a major gap between registered clinical trials and published outcomes. From 133 clinical trials registered in the US National Institute of Health–Clinical Trial database (http://clinicaltrials.gov) using MSCs from different sources to treat osteoarthritis, only six trials published their results whereas 47 trials indicated a “completed” status. On the other hand, many of the clinical trials were limited to short-term follow-ups and long-term monitoring was not conducted.

Given the limitations of MSCs in the clinical setting, EVs were proposed as an alternative to overcome these issues and increase consistency despite data limited to just nine clinical trials registered to deliver MSC-derived secretomes, exosomes and/or extracellular vesicles to osteoarthritis patients. The limited number of MSC secretome- or EV-based clinical trials demonstrated that, despite substantial interest and extensive preclinical investigation with promising results, translation to the clinic remains a distant goal.

The key differences between MSCs, MSC-derived secretome products, and MSC-derived EVs across parameters relevant to clinical translation, including efficacy, safety, scalability, manufacturing, storage, and regulatory considerations, are summarised in Table 1.

**TABLE 1:** Comparative overview of MSCs, MSC-derived secretome (conditioned medium), and MSC-derived extracellular vesicles as therapeutic strategies for osteoarthritis, across key parameters relevant to clinical translation.

Feature	MSCs	MSC-derived secretome	MSC-derived EVs
Primary therapeutic mechanism	Paracrine signalling, direct cell-cell contact, transient engraftment	Soluble factors (cytokines, growth factors, EVs combined)	miRNA, protein, and lipid cargo delivery via membrane vesicles
Efficacy in preclinical OA models	Well established; chondroprotective, anti-inflammatory, immunomodulatory	Demonstrated but variable; dependent on culture conditions	Increasingly well established; mirrors parental MSC effects
Clinical trial evidence (OA)	>133 registered trials; limited phase III data; inconsistent outcomes	Minimal; very few registered trials	∼9 registered trials; early phase only; promising but limited
Immunogenicity	Moderate; allogeneic MSCs can trigger immune responses under inflammatory conditions	Low; soluble factors generally non-immunogenic	Low; reduced MHC expression compared to parent cells
Tumorigenicity risk	Theoretical concern; genomic instability with prolonged culture	Negligible	Negligible; acellular, non-replicating
Risk of ectopic differentiation	Present; documented in preclinical models	None	None
Scalability	Moderate; limited by replicative senescence and passage-dependent potency loss	High; large volumes of conditioned medium producible	Moderate–High; 3D bioreactor systems improving yield
Manufacturing complexity	High; GMP cell expansion, quality control, release testing	Moderate; requires standardised culture and concentration steps	High; isolation, characterisation, and potency testing required
Standardisation	Challenging; donor-to-donor and lab-to-lab variability well documented	Challenging; undefined mixture of bioactive components	Improving; MISEV guidelines provide framework; potency assays needed
Storage and logistics	Cryopreservation possible but reduces potency; cold-chain dependent	Lyophilisation possible; cold-chain dependent	Cryopreservation feasible with maintained activity; off-the-shelf potential
Regulatory pathway	Advanced Therapy Medicinal Product (ATMP); complex regulatory requirements	ATMP or biological product; regulatory pathway evolving	ATMP or biological product; regulatory framework still developing
Dose definition	Cell number (typically 2–200 × 10^6^ cells)	Protein concentration or volume	Particle number or protein content; no consensus standard
Key translational barrier	Manufacturing variability, inconsistent clinical efficacy, safety concerns	Undefined composition, lack of potency assays, regulatory uncertainty	Insufficient clinical data, lack of standardised potency assays, scalable GMP production

## 3 What are EVs?

EVs, membrane-bound particles produced by most cells and secreted to the extracellular space, play an important role in cell-cell signaling. They contain cargo of lipids, proteins, miRNAs, DNA, mitochondrial fragments, etc., and are produced by a variety of cellular mechanisms including budding from the cell membrane or arising from the intracellular endosomal compartment. They are also produced by cells undergoing programmed cell death, or apoptosis. Attempts have been made to devise a nomenclature based on cellular origin and size; thus the term “exosome” was used to describe EVs ranging from 30 to 200 nm that are released from multivesicular endosomal compartments (MVB) (Simons and Raposo, 2009; Théry et al., 2009). Larger vesicles were categorized into two groups: microparticles and apoptotic bodies. Microparticles, budding from the plasma membrane, display sizes ranging from 100 nm to 1 µm (Burger et al., 2013). Larger particles ranging from 500 nm −2 µm were produced by cells undergoing apoptosis and are referred to as apoptotic bodies (Hsu et al., 2010; Battistelli and Falcieri, 2020).

**FIGURE 1:**
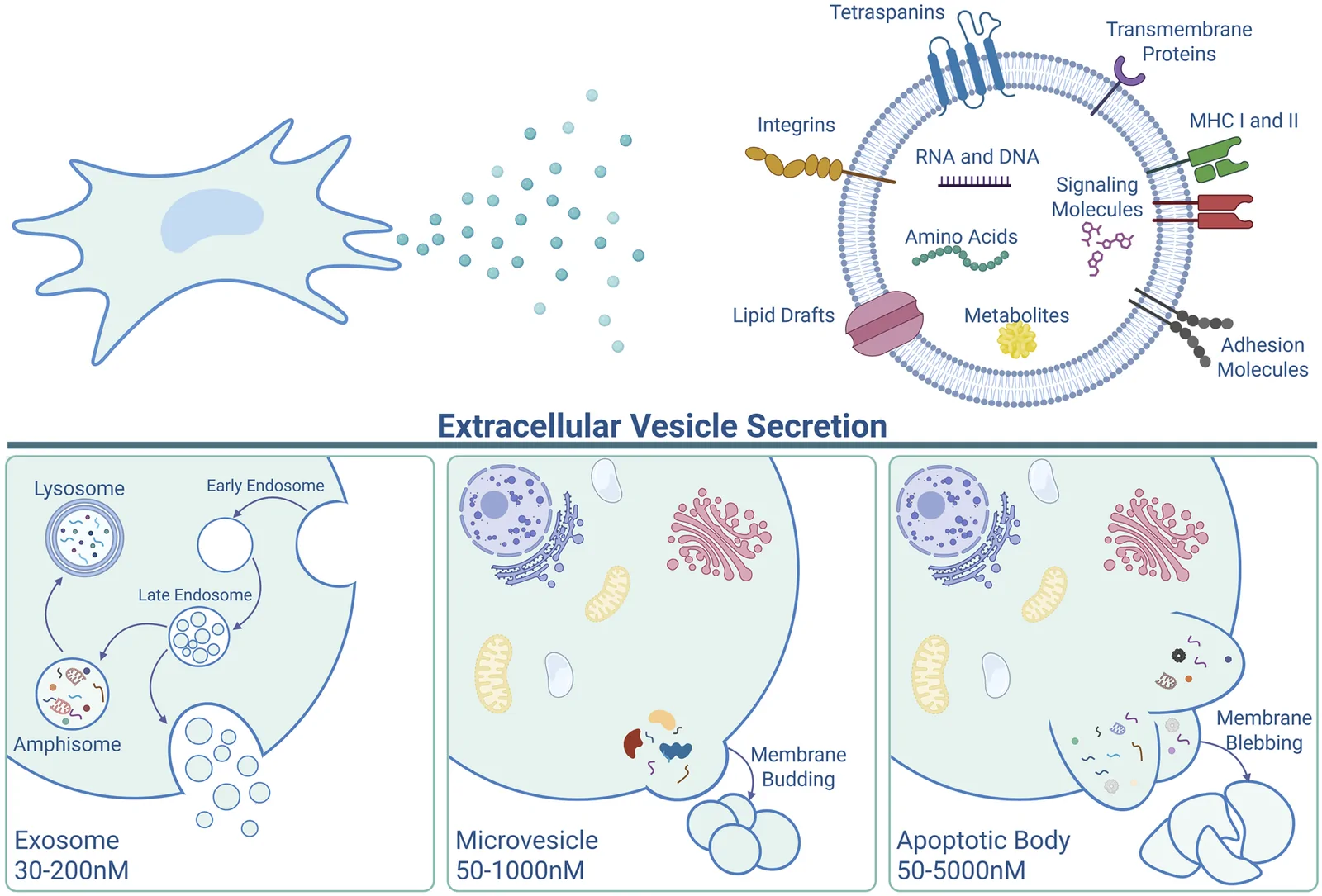
Heterogeneous mixture of EVs released by cells through three different secretion pathways. (1) Exosome form by Intraluminal vesicles formation in Late Endosomes. Late Endosomes can also form Amphisomes which degrade after fusing with lysosomes. (2) Microvesicles are released directly from the cell membrane through the membrane budding process. (3) Apoptotic bodies are produced following cell activation or apoptosis under membrane blebbing. Figure created with BioRender.com.

However, it quickly became clear that categorizing by size was not a sufficient strategy because of overlapping size distributions. As a result, classification of EVs has now been revised to reflect their biogenesis or release pathways (Colombo et al., 2014). Although the underlying mechanisms of EV production are not fully discovered, based on biogenesis pathways they are divided into three main categories of exosomes or small EVs, microvesicles (MVs) and apoptotic bodies (Figure 1). Exosomes are generated from endosomes that form multivesicular bodies (MVBs) at later stages of endosome development. Exosomes or ILVs will be carried inside these MVBs to be either degraded by lysosomes or fused with the plasma membrane to release the exosomes to the outside of the cell membrane (Kenific et al., 2021). In contrast to smaller EVs, MVs are shed from plasma membranes through a multi-stage process (Benameur et al., 2019). On the other hand, apoptotic bodies are generated under specific pathways depending on whether the cell is undergoing activation or apoptosis (Twu et al., 2014; Lawson et al., 2016; Van der Pol et al., 2012). However, uncertainty over the origin of specific EVs used in research and clinical studies is still an ongoing challenge. Therefore, characterization based on physical properties such as particle size, buoyant density, morphology and protein composition are inadequate in the definition of EV subtypes (Bobrie et al., 2011). In recent years considerable attention has been dedicated to providing a uniform definition and The International Society for EVs (ISEV) released three guidelines in 2014, 2018 and 2023 proposing minimal criteria for the study of EVs (Théry et al., 2018; Lötvall et al., 2014; Welsh et al., 2024). The MISEV guidelines provide a comprehensive framework for the nomenclature, collection, and pre-processing of extracellular vesicles (EVs), addressing critical pre-analytical variables from collection to storage. The guidelines emphasize the importance of standardisedcell culture conditions to ensure reproducibility in EV production and to enhance comparability across studies. Characterization techniques, including size distribution, marker expression, and morphology are essential to confirm the identity and purity of isolated EVs.

EVs carry a variety of lipids, proteins, and nucleic acids, including mRNA, microRNA (miR), and long non-coding RNA that play a critical role in intercellular communications, the immune response, cell signaling, proliferation, expansion and differentiation (Zhang et al., 2020). Among known EV surface markers, CD63, CD81, CD82, and CD9 are the most studied, but the expression of these in EV subtypes may vary. Despite ongoing efforts to define the composition of EVs, it is apparent that their -omics profile is heavily influenced by the isolation method, cell physiological state and the microenvironment.

## 4 The role of endogenous EVs in maintaining joint health and OA development

EVs are produced by all cells within the joint and released directly into the synovial fluid (SF): EVs from bone, chondrocytes, fibroblast-like synoviocytes (FLSs), and macrophage-like synoviocytes as well as blood-cell re-derived EVs such as leukocytes recruited from the circulation and extra-articular plasma-derived EVs are present in the joint environment (Malda et al., 2016). They all have a multitude of functions, such as maintenance of homeostasis in cartilage and controlling the bone mineralization and calcification process. In OA, multiple joint factors disturb the joint homeostasis, leading to alteration of the number and content of secreted EVs from surrounding cells (Berckma et al., 2005; Kato et al., 2014; Kolhe et al., 2017). However, during OA development, these EVs can enhance the disease progression. These changes are not only a passive reflection of disease; during OA development, the altered EV populations actively contribute to disease progression by inducing inflammatory signals, promoting cartilage catabolism, and disrupting the normal crosstalk between joint-resident cells. Understanding the specific contributions of each cell-derived EV population in both the healthy and OA joint is therefore important for defining their roles in disease pathogenesis and identifying potential therapeutic targets. The principal features of joint cell-derived EVs, including their cellular source, key cargo, target cells, and reported effects in OA, are summarised in Table 2.

**TABLE 2:** Summary of extracellular vesicles derived from joint-resident and circulating cell populations under healthy and osteoarthritic conditions, including principal cargo molecules, primary target cells, reported biological effects, and corresponding manuscript reference numbers.

EV source	Condition	Principal cargo	Target cells	Reported effects	References
Healthy joint					
Chondrocytes	Healthy	miRNAs, proteins, bioactive factors	Chondrocytes, joint cells	Synthesise and regulate ECM components; maintain hemostatic balance of matrix macromolecules; respond to mechanical stimuli; promote proliferation of naïve chondrocytes; retain cartilage-specific ECM; regulate anabolic gene expression (COL2A1, aggrecan); support cartilage-specific matrix synthesis; regulate catabolic responses and inflammatory cytokines (IL-6, TNFα); act through autophagy-dependent pathway	Akkiraju and Nohe (2015), Sophia Fox et al. (2009), Wang et al. (2023), Lee et al. (2025), Rosenthal et al. (2015)
Chondrocytes	Healthy	miR-221-3p	Osteoblasts	Contribute to bone remodelling via miR-221-3p transfer to osteoblasts; inhibit early osteogenesis markers COL1A1 and RUNX2	Shang et al. (2021)
Osteoblasts	Healthy	RUNX2, OSX, miR-34a, miR-27a, miR-22	Osteoblasts, osteoclasts	Regulate bone formation by transferring key osteogenic factors (RUNX2, OSX) and miRNAs; mediate bone resorption to prevent excessive bone formation	Narayanan et al. (2018)
Osteoclasts	Healthy	miR-214	Osteoblasts	Regulate bone homeostasis by targeting the PI3K-AKT pathway	Yuan et al. (2018), Zhao et al. (2015)
Osteocytes	Healthy	Sclerostin, RANKL, osteoprotegerin, bone regulatory proteins	Osteoblasts, osteoclasts	Respond to mechanical stimulation and Ca^2+^ oscillation; promote osteogenic differentiation and mineralisation; promote adaptive bone remodelling and mineralisation in response to joint loading	Morrell et al. (2018), Nieuwoudt et al. (2021), Solberg et al. (2015)
Bone marrow-derived MSCs	Healthy	Not specified	Macrophages	Modulate resting macrophages in the joint; induce phenotypic polarisation to promote tissue regeneration	Chen et al. (2020a)
Fibroblast-like synoviocytes (FLS)	Healthy	miR-126-3p, miR-150-3p, miR-214-3p, miR-4738-3p, hyaluronic acid (HA)	Chondrocytes, joint-resident cells	Maintain anti-inflammatory conditions; modulate chondrocyte and joint-resident cell phenotypes; suppress joint degradation; support cartilage matrix synthesis (COL2A1, ACAN); increase chondrocyte proliferation; inhibit pro-inflammatory responses; enhance anabolic signals; reduce inflammatory cytokines; contribute to metabolic maintenance and joint nutrition	Zhou et al. (2021), Wang et al. (2022), Lai et al. (2023), Xu et al. (2024), Smith and Ghosh (1987)
Macrophage-like synoviocytes (MLS)	Healthy	Anti-inflammatory factors (inferred; M2-like phenotype)	Joint-resident cells	Promote tissue repair and clearance of debris; regulate immune homeostasis; recycle lubricin	Menarim et al. (2020), Kurowska-Stolarska and Alivernini, (2017)
Platelets	Healthy	bFGF, VEGF, PDGF-BB, TGF-β1, bioactive molecules	Chondrocytes, joint-resident cells	Support tissue repair and regenerative processes in the joint upon activation	Hou et al. (2023)
Leukocytes/blood-derived EVs	Healthy	Anti-inflammatory factors	Chondrocytes, joint-resident cells	Maintain balanced signalling; help resolution of minor injuries; protect chondrocytes by preventing inflammatory cytokine production	Otahal et al. (2020)
OA Joint					
Chondrocytes (OA cartilage)	OA	Alkaline phosphatase, BMP	Subchondral bone, periarticular tissue	More potent in initiating *in vitro* calcification with increased alkaline phosphatase activity; premature chondrocyte maturation leads to release of matrix EVs with higher BMP levels, stimulating subchondral bone overgrowth and periarticular osteophyte formation	Ali and Wisby, (1978), Einhorn et al. (1985), Nahar et al. (2008), Stein et al. (1981)
Chondrocytes (IL-1β stimulated)	OA	miR-449a-5p	FLS, macrophages, chondrocytes	Increase MMP-13 production in FLS; promote IL-1β production in macrophages; miR-449a-5p inhibits ATG4B suppressing autophagy and leading to chondrocyte apoptosis; inhibition of miR-449a shown to restore cartilage and prohibit OA progression	Nakasa et al. (2012), Ni et al. (2019), Ni et al. (2019), Baek et al. (2018), Nakasa et al. (2012), Ni et al. (2019), Baek et al., (2018)
Chondrocytes (TMJ-OA)	OA	Not specified	Cartilage matrix	EV numbers correspond to degree of matrix calcification in TMJ-OA model induced by unilateral anterior crossbite (UAC); inhibition of chondrocyte-derived exosome formation eliminated abnormal calcification	Liu et al. (2022)
Senescent chondrocytes	OA	SASP factors; miR-34a, miR-30c, miR-125a, miR-24, miR-92a, miR-150, miR-186 (altered by senolytic treatment); miR-27b, miR-199a, miR-185 (senescence-specific, absent in age-related degeneration)	Non-senescent chondrocytes, subchondral bone	Higher EV numbers after senescence; transfer senescence factors to healthy chondrocytes inhibiting ECM formation; SASP elevates matrix-degrading enzymes and pro-inflammatory cytokines (IL-6, IL-1α, MCP-1); senescence-specific miRNAs cause abnormal subchondral bone development, dysregulated chondrogenesis, and catabolic and inflammatory gene expression	Misawa et al. (2020), Tsuchida et al. (2014), Jeon et al. (2019)
Osteoclasts	OA	TIMP-2 and TIMP-3 inhibitors	Chondrocytes, osteoblasts	Facilitate crosstalk between chondrocytes and osteoclasts; promote chondrocyte hypertrophy and degeneration; damage accelerated by TIMP-2 and TIMP-3 inhibition; miRNA profile changes at earlier OA stages indicate cargo role in disease progression	Han, (2022), Lei et al. (2021)
Osteoblasts	OA	miR-210-5p	Chondrocytes	Cause subchondral bone sclerosis; trigger catabolic activity of chondrocytes; negatively affect cartilage metabolism	Wu et al. (2021)
Fibroblast-like synoviocytes (FLS)	OA	TNF-α, IL-1β, ECM-degrading enzymes	Joint cells, ECM	Contribute to persistent inflammation and joint destruction; produce pro-inflammatory cytokines TNF-α and IL-1β and enzymes leading to ECM degradation	Ding et al. (2015), Wang et al. (2006), Noss and Brenner, (2008)
Fibroblast-like synoviocytes (FLS) — IL-1β stimulated	OA	ADAMTS-5, MMP-13 (increased); COL2A1, ACAN (decreased)	Articular chondrocytes	IL-1β stimulation drives FLS to release EVs that increase ADAMTS-5 and MMP-13 and decrease COL2A1 and ACAN in chondrocytes	Kato et al. (2014)
Fibroblast-like synoviocytes (FLS) — RA context	RA	Citrullinated proteins, TNF-α membrane form	Joint cells	EVs contain citrullinated proteins and a membrane form of TNF-α, reported in RA patients	(Skriner et al., 2006; Zhang et al., 2021a)
Fibroblast-like synoviocytes (FLS)	OA	LncRNA H19	Chondrocytes	LncRNA H19 targets miR-106b-5p/TIMP2 axis; promotes chondrocyte proliferation and migration; inhibits degradation of joint matrix	Tan et al. (2020)
Synoviocytes	OA (late stage)	LncRNA-PCGEM1	Synoviocytes	Higher levels observed in later OA stages; acts as sponge for miR-770, a regulator of proliferation, apoptosis and autophagy	Kang et al. (2016)
Synoviocytes — gender-specific	OA	Gender-specific miRNA cargo	Joint cells	Contrasting miRNA profiles between male and female OA patients in synovial fluid; suggests a role for estrogen in EV-derived miRNA regulation in female knee joints	Kolhe et al. (2017)
Synovial fibroblasts (hypoxia — RA context)	RA	miR-424	T cells	EVs released under hypoxia enriched in miR-424; induce T cell differentiation and contribute to escalation of rheumatoid arthritis	Ding et al. (2020)
Synovial fluid EVs (mixed origin)	OA	Peptides reflecting OA severity	Joint cells	Size and concentration of SF EVs unchanged compared to non-OA individuals; specific surface markers, origins and effects remain undiscovered; carry peptides directly reflecting severity of OA knee radiographic changes	Withrow et al. (2016), Zhang et al. (2023)
Platelets (RA context)	RA	Platelet-derived cargo	Joint cells	Platelet-derived EVs found in synovial fluid of RA patients but not OA patients; delivery to the joint mediated by neutrophils after internalisation	Boilard et al. (2010), Duchez et al. (2015)
Plasma-derived EVs	OA	TNF-α	Joint cells	Deliver high volumes of TNF-α to the joint, contributing to OA damage	Kolhe et al. (2017)
Platelets (OA serum)	OA	Annexin V+, annexin V+ CD45^−^ CD61^+^ particles	Joint cells	Elevated in OA patient serum compared to normal sera; potential biomarker utility	Michael et al. (2019)
Platelet-poor plasma (PPP)-derived EVs	OA (early vs. late)	CD56, ROR1, CD326 (early OA); CD63, CD31 (late OA)	Joint cells	Surface marker profile shifts between early and late OA stages; proposed as useful biomarker tool to distinguish disease stage	Matejova et al. (2023)
Serum-derived EVs	OA	LncRNA PVT1 (elevated), miR-193b (reduced)	Chondrocytes	Higher LncRNA PVT1 and lower miR-193b in OA patient serum vs. healthy donors; LncRNA PVT1 regulates chondrocyte apoptosis in OA cartilage; miR-193b regulates chondrogenesis and chondrocyte metabolism; precise effects of these changes in the joint remain unclear	Meng et al. (2020), Meng et al. (2018), Li et al. (2017)
T cells	OA	CD3^+^CD4^+^ markers	Joint cells, synovium	Elevated CD3^+^CD4^+^ in EVs from OA patient blood corresponding to inflammatory features of T cells in OA development	Oba et al. (2019)
T cells and monocytes (TNF-α treated)	OA	Arachidonic acid; COX-2 and mPGES-1 upregulation	FLS	Increased PGE2 production via upregulation of COX-2 and mPGES-1; transport of arachidonic acid from leukocytes to fibroblasts for PGE2 production	Jüngel et al. (2007)
T cells and B cells	OA	Altered cargo (not specified)	Joint cells	Altered T and B cell profiles in OA patients reflected in secreted EVs, contributing substantially to OA pathogenesis	Zhu et al. (2020)
Macrophages (M1)	OA	Pro-inflammatory cargo	Chondrocytes	Augment chondrocyte catabolism, contributing to cartilage degradation	Ebata et al. (2023)
Macrophages (M2 polarised)	OA	Anti-inflammatory cargo	Macrophages	M2 polarised EVs alleviate OA through polarisation of inflammatory macrophages in the joint	Qin et al. (2023)

BMP, bone morphogenetic protein; ECM, extracellular matrix; FLS, fibroblast-like synoviocytes; MLS, macrophage-like synoviocytes; MMP, matrix metalloproteinase; ADAMTS, a disintegrin and metalloproteinase with thrombospondin motifs; SASP, senescence-associated secretory phenotype; PGE2, prostaglandin E2; TMJ, temporomandibular joint; HA, hyaluronic acid; RA, rheumatoid arthritis; PPP, platelet-poor plasma; UAC, unilateral anterior crossbite.

### 4.1 EVs in healthy joint

#### 4.1.1 Cartilage and bone derived EVs

Chondrocytes are vital in maintaining joint health. They synthesise and regulate major ECM components, maintain a hemostatic balance of matrix macromolecules and respond to mechanical stimuli to with adopting and optimizing cartilage durability, by producing EVs into joint environment (Akkiraju and Nohe, 2015; Sophia Fox et al., 2009; Wang et al., 2023). Chondrocyte derived EVs deliver key components to other chondrocytes as well as other joints cells. These EVs contain microRNAs (miRNAs), proteins, and other bioactive factors that help proliferation of naïve chondrocytes, retention of cartilage-specific extracellular matrix (ECM) and regulation of anabolic gene expression (COL2A1 and aggrecan, for example). They support the synthesis of cartilage-specific matrix and regulate catabolic responses and inflammatory cytokines such as IL6 and TNFα (Lee et al., 2025). Chondrocyte-derived EVs primarily target other chondrocytes involved in joint regulation and cartilage metabolism through an autophagy-dependent pathway (Rosenthal et al., 2015). These EVs contribute to bone remodeling through miR-221-3p transfer to osteoblasts and inhibition of the early osteogenesis markers COL1A1 and RUNX2 (Shang et al., 2021). Similar to chondrocytes, bone-derived EVs are specialized in carrying multiple components in the healthy joint and facilitating bone and joint communication, in particular under mechanical loading. Osteoblast-derived EVs regulate bone formation by transferring key factors involved in osteogenesis such as (RUNX2 and OSX) and miRNAs (miR-34a, miR-27a, and miR-22) (Narayanan et al., 2018). On the other hand, these EVs also mediate bone resorption to prevent excessive bone formation. For example, osteoclast-EVs are enriched in miR-214 which is vital in regulating bone homeostasis by targeting the PI3K-AKT pathway (Yuan et al., 2018; Zhao et al., 2015). Under mechanical loading, osteocytes as mechanosensitive cells respond to mechanical stimulation and Ca^2+^ oscillation activation which enables EV production from these cells (Morrell et al., 2018). Osteocyte-derived EVs contain bone regulatory proteins and promote osteogenic differentiation and mineralization (Nieuwoudt et al., 2021). EVs containing sclerostin, RANKL, osteoprotegerin, and other regulators that promote adaptive bone remodeling and mineralization in response to joint loading are released (Morrell et al., 2018; Solberg et al., 2015). EVs from bone marrow-derived MSCs also modulate resting macrophages in the joint; the EVs are recruited and induction of their phenotypic polarization can promote tissue regeneration (Chen et al., 2020a).

#### 4.1.2 Fibroblast-like synoviocyte and macrophage-like synoviocytes derived EVs

The synovial membrane exhibits a unique cellular lining of two main types of cells 1) fibroblast-like synoviocytes (FLS) and 2) macrophage-like synoviocytes (MLS). FLS produces key synovial fluid components for the articular joint, like cytokines, tissue components and nutrients (Bartok and Firestein, 2010). Although the origin of FLS is unknown, it is postulated that they are MSCs of non-joint origin because of their similarity with MSCs in terms of morphology and surface markers expression (Bartok and Firestein, 2010). MLS are bone marrow-derived macrophages with very similar characteristics to other tissue-resident macrophage populations. These cells clear debris from the joint environment by phagocytosis and act as front-line defenders in response to microbial exposure (Edwards and Willoughby, 1982; Henderson and Pettipher, 1985). FLS-derived EVs maintain anti-inflammatory conditions within joints and modulate the chondrocytes and other joint-resident cell phenotypes. Also, they suppress joint degradation with supporting cartilage matrix synthesis (e.g., COL2A1 and ACAN), increasing chondrocyte proliferation. By carrying microRNAs such as miR-126-3p (Zhou et al., 2021), miR-150-3p (Wang et al., 2022), miR-214-3p (Lai et al., 2023), and miR-4738-3p (Xu et al., 2024), their EVs inhibit pro-inflammatory responses and enhance anabolic signals and reduce inflammatory cytokines from chondrocytes. Their contribution in metabolic maintenance and providing nutrition like hyaluronic acid (HA) in joints enhance cartilage health (Smith and Ghosh, 1987). MLS in healthy joint represents an M2-like phenotype (Menarim et al., 2020) which secrete EVs that promote tissue repair and clearance of debris. Although there is no particular study focusing on MLS-derived EVs in the healthy joint, because of their similar characteristics to M2 macrophages it can be anticipated that their EVs are crucial in communicating with other cells and regulating immune homeostasis and recycling lubricin (lubricating components of synovial fluid) (Kurowska-Stolarska and Alivernini, 2017).

#### 4.1.3 Blood-derived EVs in the joint

Other immune cells (lymphocytes, mast cells and dendritic cells) are scarce in the normal synovium and localize mainly in perivascular areas of the sublining layer of joints. Leukocytes, erythrocytes and platelet derived EVs from plasma serve an essential role in communication between resident cells in the joint and circulatory systems. In healthy tissue, leukocyte derived EVs maintain balanced signaling and help resolution of minor injuries by carrying anti-inflammatory factors. Platelet-derived EVs release growth factors (such as bFGF, VEGF, PDGF-BB, TGF-β1) and bioactive molecules upon activation, supporting tissue repair and regenerative processes in the joint (Hou et al., 2023). Blood derived EVs have been shown to have protective effect on chondrocytes by preventing inflammatory cytokines production (Otahal et al., 2020).

### 4.2 EVs in OA joint

#### 4.2.1 Cartilage and bone derived EVs

EVs isolated from articular cartilage of OA patients were more potent in initiating *in vitro* calcification with increased alkaline phosphatase activity (Ali and Wisby, 1978; Einhorn et al., 1985). Premature maturation of chondrocytes in OA leads to the release of matrix EVs with higher levels of bone morphogenetic protein (BMP) that stimulate subchondral bone overgrowth and periarticular osteophyte formation (Nahar et al., 2008; Anderson, 2003; Stein et al., 1981). To understand the role of chondrocyte-derived EVs in OA, IL-1β can be used to mimic the disease environment, as one of the key components released by inflammatory cells such as macrophages. EVs released by chondrocytes treated with IL-1β increased matrix metalloproteinase-13 (MMP13) production in fibroblast-like synoviocytes and as well as promotion of IL-1β production in macrophages (Nakasa et al., 2012; Ni et al., 2019; Ni et al., 2019). It has been suggested that these EVs contain miR-449a-5p, which inhibits ATG4B, one of the autophagy-related genes. ATG4B involves macro-autophagy in the healthy joint and is vital for endochondral homeostasis and cell survival. Their inhibition can lead to autophagy inhibition and chondrocyte apoptosis. This confirms a previous finding indicating the impact of miR-449a inhibition on cartilage restoration and prohibition of OA progression (Ni et al., 2019; Baek et al., 2018). In addition, the number of EVs released by chondrocytes in the temporomandibular joint (TMJ) using an OA model induced by unilateral anterior crossbite (UAC) corresponded to the matrix calcification. The abnormal calcification was eliminated after inhibiting the chondrocyte derived-exosome formation, emphasizing the role of these EVs in OA development (Liu et al., 2022). Senescent-associated secretory phenotype (SASP) factors are crucial components in development of aging-associated disorders such as OA (Misawa et al., 2020). SASP in OA cartilage elevates the matrix-degrading enzymes levels and proinflammatory cytokines like IL-6, IL-1α and monocyte chemoattractant protein 1 (MCP-1) (Tsuchida et al., 2014). Therefore, secreted EVs from arthritic cartilage derived chondrocytes showed higher numbers after senescence. Senescence factors were transferred through these EVs to non-senescent chondrocytes, leading to inhibition of ECM formation by healthy chondrocytes. The expression of miRs such as miR-34a, −30c, −125a, −24, −92a, −150 and −186 is altered by senolytic treatments on synovial fluid exosomes, which suggests that senescence-derived EVs are responsible for controlling OA development. The progression of the disease is also facilitated by miRs like miR-27b, miR-199a and miR-185, which are present in senescence associate EVs but not in age-related degenerated EVs, again indicating their mechanism of action by causing abnormal subchondral bone development, chondrogenesis, and catabolic and inflammatory gene expression (Jeon et al., 2019). Also, bone derived EVs affect the joint environment contributing to cartilage degradation and bone remodeling. During OA development osteoclasts facilitate crosstalk between chondrocytes and osteoclasts and promote chondrocyte hypertrophy and degeneration. It has also been suggested that this damage can be accelerated by TIMP-2 and TIMP-3 inhibition (Han, 2022). At earlier stages of OA, the miRNA profile of these EVs undergoes changes indicating their cargo role in disease progression (Lei et al., 2021). Osteoblast-derived EVs have a negative role in cartilage metabolism also, causing subchondral bone sclerosis by carrying miRNAs such as miR-210-5p and triggering catabolic activity of chondrocytes (Wu et al., 2021).

#### 4.2.2 Fibroblast-like synoviocyte (FLS) and macrophage-like synoviocyte derived EVs

Inflammatory and catabolic factors released by FLS contribute to persistent inflammation and destruction of joints in OA (Ding et al., 2015). They produce proinflammatory cytokines such as TNF-α and IL-1β, and enzymes that lead to extracellular matrix (ECM) degradation (Wang et al., 2006; Noss and Brenner, 2008). Treatment with IL-1β stimulates synovial fibroblasts (SFB) to release EVs, leading to increased ADAMTS-5 and MMP-13 expression and decreased COL2A1 and ACAN in articular chondrocytes (Kato et al., 2014). FLS-derived EVs were reported to contain citrullinated proteins, a TNF-α membrane form found in rheumatoid arthritis (RA) patients (Skriner et al., 2006; Zhang et al., 2021a). Tan et al. also investigated the role of FLS-derived exosomes in chondrocyte regulation. LncRNA H19 in these EVs targets miR-106b-5p/TIMP2 and promoted chondrocytes proliferation and migration as well as inhibiting degradation of the joint matrix in OA (Tan et al., 2020). Higher levels of EVs containing lncRNA-PCGEM1 were observed in the later stages of OA, which acts as a sponge for synoviocyte miR-770, a regulator for proliferation/apoptosis and autophagy (Kang et al., 2016). Kolhe and co-workers observed a different level of exosomal miRNA expression in synovial fluid of OA patients from different genders. Interestingly, the miR cargo of these EVs showed a contrasting profile in male compared to female patients suggesting a role for estrogen in EV-derived miRNA in female knee joints (Kolhe et al., 2017). In rheumatoid arthritis, synovial fibroblast-derived exosomes released under hypoxia were enriched in miR-424. EVs containing miR-424 induced differentiation of T cells and contributed to the escalation of rheumatoid arthritis (Ding et al., 2020). Both synoviocytes and chondrocytes act as the source of EVs released to the SF. Despite the contribution of secreted EVs from these cells in OA development, both size and concentration of the SF EVs remained unchanged in comparison to non-OA individuals (Withrow et al., 2016). Although the role of synovial fluid-EVs in OA pathogenesis has been widely investigated, their specific surface markers, origins and effects are still undiscovered. It has been suggested that SF EVs carry peptides that are directly reflect the severity of OA knee radiographic (Zhang et al., 2023).

#### 4.2.3 Blood-derived EVs

Synovial Fluid is enriched by diverse plasma derived EVs sourced from blood cells and other tissue-derived cells in the circulation. In contrast to OA patients, synovial fluid of patients with RA contained platelet-derived EVs (Boilard et al., 2010). Their delivery to the joint was shown to be mediated by neutrophils after internalization (Duchez et al., 2015). Plasma derived EVs are also responsible for delivering high volumes of TNF-α to the joint and thus contributing to OA damage (Kolhe et al., 2017). As direct analysis of the plasma-derived EVs in an affected joint is challenging, circulating blood-derived plasma can act as a suitable source to study changes of EVs derived from plasma in healthy and OA patients at different stages. Platelet-derived EVs in OA patient’s serum, such as annexin V^+^ and annexin V^+^ CD45^−^ CD61^+^ EVs were higher than in normal sera (Michael et al., 2019). Also, by comparing two sources of plasmas (OA patients and healthy donors), it has been suggested that the components of platelet-poor plasma (PPP)-derived EVs can be a useful biomarker tool. In early OA patients, CD56, ROR1 and CD326 were expressed more in these EV, while in the later stages of OA, EVs expressed more CD63 and CD31 (Matejova et al., 2023). The content of these EVs were also affected in OA patients. For example, serum EVs of patients had higher levels of LncRNA PVT1 and lower expression of miR-193b compared to healthy donors (Meng et al., 2020; Meng et al., 2018). Interestingly, LncRNA PVT1 in OA cartilage can regulate chondrocyte apoptosis and miR-193b regulates chondrogenesis and chondrocyte metabolism (Meng et al., 2018; Li et al., 2017). However, despite the alteration that was observed in plasma EVs quantity and quality, the precise effects of these changes in the joint are not clear.

T-cells can be considered as another source of EVs in SF. Similar to plasma, specific immune cell-derived EVs cannot be directly studied in the OA joint. As such, serum from OA patients can be substituted as a source in this respect. EVs from T-cells derived from OA patient blood are reported to contain elevated levels of CD3^+^CD4^+^, corresponding to inflammatory features of these cells in OA development (Oba et al., 2019). In another study, Jungel et al. investigating EVs derived from TNF-α-treated T cells and monocytes showed increased production of PGE2 by upregulating COX‐2 and mPGES‐1; transport of arachidonic acid from leukocytes to fibroblasts for PGE2 production was also observed (Jüngel et al., 2007). Additionally, the profile of T cells and B cells of OA patients were shown to be altered which might be reflected in secreted EVs produced by these cells contributing substantially to the pathogenesis of OA (Zhu et al., 2020). Macrophages as one of the key players in both normal and OA joints can be considered as the source of extracellular vesicles. These EVs augment chondrocyte metabolism, contributing to cartilage degradation (Ebata et al., 2023), while M2 polarized EVs alleviated OA through polarization of inflammatory macrophages in the joint (Qin et al., 2023).

## 5 The role of MSC-derived extracellular vesicles in osteoarthritis treatment mirrors that of their parent cells

**FIGURE 2:**
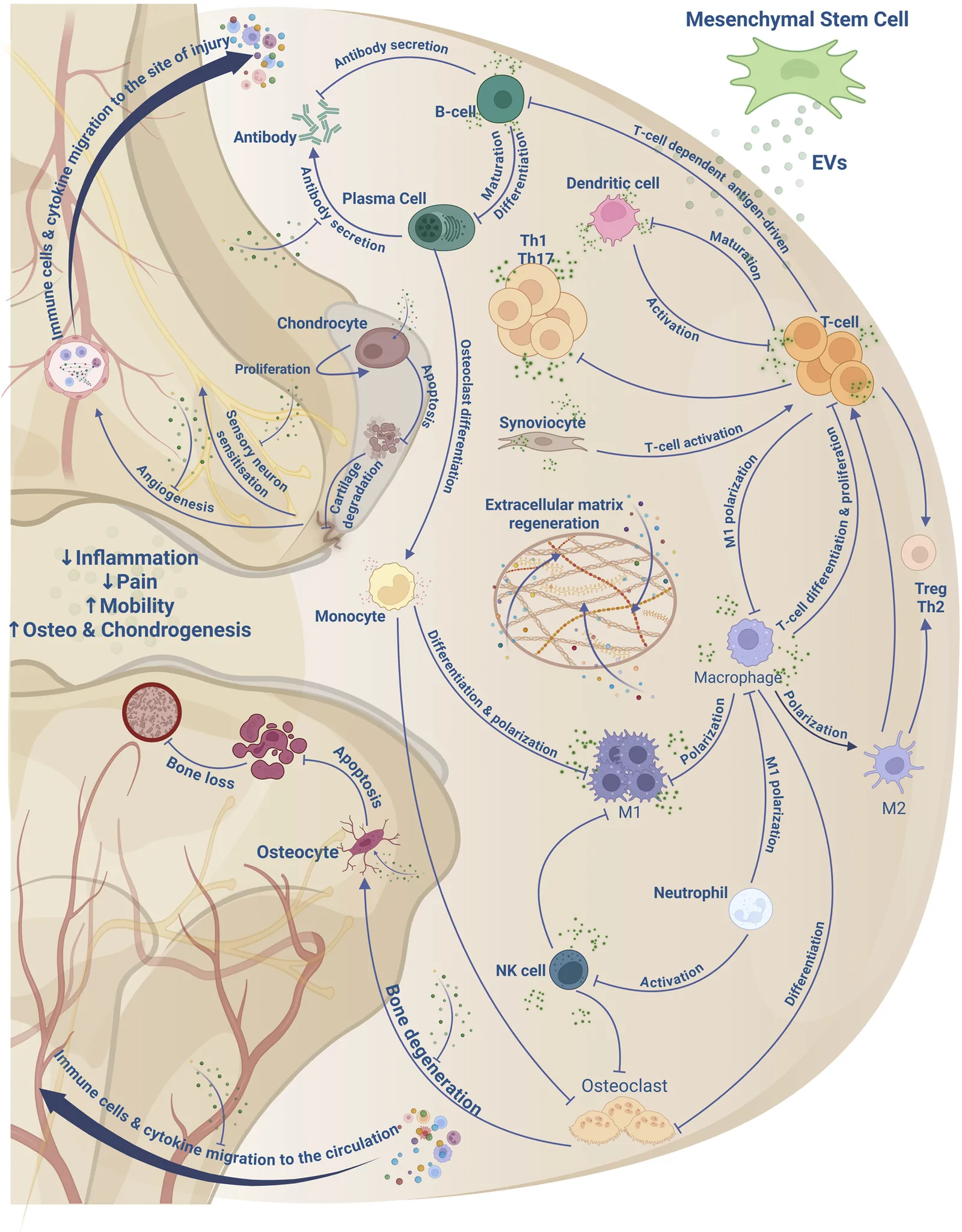
Schematic representation of the multifaceted roles of MSC-derived extracellular vesicles (MSC-EVs) on different cell populations within the osteoarthritic joint. Following intra-articular administration, MSC-EVs interact with multiple joint-resident and infiltrating cell types to exert their therapeutic effects. On chondrocytes: MSC-EVs promote chondrocyte proliferation and migration, enhance the synthesis of cartilage extracellular matrix components (including Collagen type II and aggrecan), inhibit catabolic enzymes (MMP-13, ADAMTS-5), and suppress chondrocyte apoptosis. These effects are mediated in part through the delivery of chondroprotective miRNAs and activation of pro-survival signalling pathways. On macrophages: MSC-EVs suppress M1 pro-inflammatory macrophage activation and promote polarisation toward the M2 anti-inflammatory phenotype, reducing the production of inflammatory cytokines, while increasing anti-inflammatory cytokines. This shift in macrophage phenotype reduces synovial inflammation and creates a more permissive environment for tissue repair. On T cells and other immune cells: MSC-EVs suppress T-cell proliferation, promote regulatory T-cell (Treg) expansion, and inhibit B-cell activation and antibody production, collectively dampening the adaptive immune response that contributes to OA progression. On osteocytes and subchondral bone: MSC-EVs modulate osteoblast and osteoclast activity, reducing pathological subchondral bone remodelling and osteophyte formation. At the joint level: The combined effect of MSC-EV administration is a reduction in synovial inflammation, attenuation of cartilage ECM degradation, inhibition of subchondral bone sclerosis, and amelioration of joint pain. These effects are mediated through a complex network of EV cargo molecules including miRNAs, proteins, and lipids that act in a coordinated, multi-target fashion across the joint microenvironment. Figure created with BioRender.com.

Experimental evidence investigated the role of MSCs-derived EVs in both *in vitro* and *in vivo* OA models. These EVs are similar to their parental cells and exhibit shared features in OA environments. The therapeutic effects of MSC-EV in OA are multidimensional and can be grouped into three major mechanisms of actions: chondroprotection, immunomodulation and regulation of subchondral bone remodeling (Figure 2). The evidence reviewed in subsequent sections indicates that MSC-EVs from a single source can simultaneously exert all three of these effects, reflecting the multifunctional cargo of miRNAs, proteins, and lipids they carry. However, the relative dominance of each effect may vary by source.

### 5.1 Remarkable *in vivo* findings of the role of MSC-EVs in OA

The fundamental of the mechanisms of MSC-EVs has been proven using *in vitro* studies; however, *in vivo* findings in animal models of OA have further validated their potential by showing that MSC-EVs can effectively decelerate the degradation of cartilage, reduce inflammation and promote tissue regeneration in injured joints.

As described, MSCs derived EVs in OA treatment play a multi-dimensional therapeutic role. Numerous studies evaluated these EVs in animal models to determine their ultimate outcome and assess their clinical potential. For instance, in a study involving OA rats, 6–12 weeks injection of human ESC derived MSC-exosomes demonstrated significant repairing capability in treating the cartilage of critical-sized defects. The treated rats exhibited joint regeneration with uniform and intense glycosaminoglycan (GAG) staining with sufficient levels of hyaline cartilage and Col II and underlying subchondral bone (Zhang et al., 2016). Subsequent research by Zhang further explored the underlying multi-faceted mechanism of MSC exosome-mediated cartilage regeneration. In this study, exosomes enhanced surface uniformity and integration with existing cartilage, with higher Col II and Col VI levels (Zhang et al., 2018a). They also demonstrated human MSC-derived exosome effects on temporomandibular joint osteoarthritis (TMJ-OA) in a rat model with OA induced by monosodium iodoacetate. MSC derived exosomes reduced the gene expression of pro-inflammation gene expression, apoptosis, fibrosis, and with decreased pain scoring (Zhang et al., 2019). In another study, mice treated with an injection of hESC-MSC exosome (1 × 10^6^ particles per joint) showed chondroprotective effects 4 weeks after the treatment. Injection of these EVs into mice exposed to destabilization of the medial meniscus (DMM), decreased deconstruction of cartilage ECM was observed 4 weeks after treatment (Wang et al., 2017a). Mice MSC-derived exosomes and microparticles showed a similar chondroprotective effect in the OA animal model. Administration of the exosomes improved cartilage volume and thickness compared to the control group (Cosenza et al., 2017). Zhu and his colleges compared the OA treatment potency of exosomes released from iPSC derived MSCs and synovial membrane-derived MSCs (SMMS). In this study, six-week-old female mice were injected with collagenase to generate an OA model. Exosomes treatment on days 7, 14, and 21 exhibited higher levels of cartilage regeneration in the iMSC group compared to SMMS (Zhu et al., 2017).

MSC engineering has also been used to enhance their therapeutic potential by incorporating various miRNAs. Exosomes from MSCs overexpressing miR-92a-3p inhibited severe cartilage damage in collagen-induced OA mice model (Mao et al., 2018). On the other hand, exosomes secreted by miR-140-5p overexpressing human SMMSC (10^11^ particles/mL) were injected into an OA rat model. This study aimed to overcome one of the SMSC exosome drawbacks: inhibition of SOX9 and ECM generation by inducing 140-5p overexpression in these MSCs. This prevented the reduction of chondrocyte levels resulting in an improved OARSI score (Tao et al., 2017). Preconditioning of rat MSCs with TGF- β1 treatment led to exosome production with higher miR-135b levels. Injecting of 10^11^ EVs/mL into the 12-week-old male rats enhanced the number of chondrocytes, and reduced OARSI score compare to control groups (Wang et al., 2018). Exosomes overloaded with MiR-100-5p secreted from human infrapatellar fat pad MSCs (IPFP-MSC) prevented cartilage tissue destruction and improved OA pain in gait analysis. Administration of human MSC derived exosomes overexpressing KLF3-AS1 in the rat OA model decreased Mankin scores and hypertrophy markers (Liu et al., 2018a). To generate an *in situ* formed acellular hydrogel glue tissue patch, MSCs derived from human iPSC-produced exosomes were combined with a PIC hydrogel. This patch mediated exosome delivery and improved the formation of a well-organized articular cartilage structure (Liu et al., 2017). MSC EVs in combination with 3D printed cartilage extracellular matrix (ECM)/gelatin methacrylate (GelMA) as a scaffold regenerated hyaline cartilage with an improved ICRS score. This bio-scaffold offered a new material for restoring of cartilage in a rabbit OA model (Chen et al., 2019). In another study, umbilical MSCs were expanded on hollow-fiber culture cartridges to mimic the 3D structure, and their EV role in cartilage defects was evaluated in the rat model. However, defects treated with EVs from both 2D and 3D MSC culture conditions partly filled with hyaline cartilage 3D-EVs exhibited better thickness with the highest Wakitani score (Yan and Wu, 2019). Comparing the MSC-EVs and another type of platelet-derived EVs (pEVs) in an OA cartilage rat model demonstrated interesting results; platelet-derived EVs showed superior effects on OA reversion compared to MSC- derived EVs, with particular improvement in female knee OA rat models (Forteza-Genestra et al., 2024). Rat models of OA treated with miR-29a-loaded vesicles improved the stability of cartilage extracellular matrix, suggesting the role of this microRNA in controlling the level of inflammatory factors and apoptosis similar to other findings (Yang et al., 2024; Dey et al., 2021). MSC-derived EVs in collagenase-induced OA model were functionally impaired and unable to mitigate the cartilage damage (Boulestreau et al., 2024). Conferring effect of SF-MSCs derived EVs (25 and 50 µg of EVs) compared to hyaluronic acid in OA model was observed by increased anabolic markers and decreased catabolic markers. Their anti-inflammatory effects were not limited to the joint and were systematic with significant reduction of pro-inflammatory cytokine level in serum (Liang et al., 2023). Increased osteophyte volume is a key diagnostic feature of OA; in surgical rat models of OA treated with human-(BM)-MSC derived EVs (7.77 × 10^7^ particles) derived from 2 × 10^6^ MSCs compared to MSCs alone, MSC treatment induced osteophyte generation while MSC-EV treatment diminished the differing results (Warmink et al., 2023). However, MSCs can be primed with chondrocyte co-culture and mimic the joint environment after injection. EVs, derived from this condition and used in a monoiodoacetate (MIA) rat OA model, showed improved and matrix production. This improvement was manifested by increasing the ratio of chondrocytes to MSCs in culture that might highlight the importance of preconditioning of MSCs prior to EV collection for higher recovery (Hosseinzadeh et al., 2023). LPS-preconditioned MSCs derived EVs also improved DMM-induced inhibited reduction of COL2A1 and aggrecan and accumulation of ADAMTS5 caused by IL-1β (Duan et al., 2021). The effect of EVs from umbilical cord derived MSC on the OA mice knee revealed the possible interaction of miR-1208 with MERRL3 leading to reduction of M6A in NLRP3 of macrophages and subsequently reducing M1 pro-inflammatory effects through IL-1β (Zhou et al., 2022). In another study, EVs injected into OA joints created by the surgical transection of the anterior cruciate ligament (ACLT), ameliorated the disease progression. This study highlighted that this effect was through M2 macrophage polarization *in vitro* (Li et al., 2022a). EVs from circHIPK3 overexpressing MSCs increased PCNA-positive cells in an OA model with apoptosis inhibition in chondrocytes via the miR-124-3p/MYH9 axis (Li et al., 2021). NEAT1 as one of the key factors in the innate immune system response was also detected to be transferred via BM-MSC-EVs to chondrocytes in an *in vivo* model. After binding to miR-122-5p, NEAT1 leads to an increase in Sesn2 levels, inhibiting Nrf2 pathway activation and improving proliferation and autophagy of chondrocytes but also apoptosis inhibition (Zhang and Jin, 2022). Hypoxic pre-conditioning was also shown to alter MSC-EV miR profile and positively impact their effect on the OA model. Bioinformatic analysis of these EVs revealed the role of MSC-EVs on miRNA-18-3P/JAK/STAT or miRNA-181c-5p/MAPK signaling pathways, suppressing chondrocyte apoptosis and stimulating their proliferation and migration (Zhang et al., 2022a). Also, Wharton’s jelly derived MSCs (WJMSCs) carried chondroprotective-related microRNAs such as let-7e-5p. Here, suppression of STAT3 and IGF1R enhanced articular cartilage repair in rat models after anterior cruciate ligament transection (ACLT) (Chen et al., 2022).

The frequency of treatment is one of the major challenges in the OA models studied. In a systematic review by Zhang et al. comparing *in vivo* studies administering MSC-EVs, he found that these EVs exhibited more efficacy at earlier stages of OA and administration of once a week showed better results than multiple times a week (Zhang et al., 2021b). The findings from *in vivo* studies highlight the considerable potential of MSC-EVs as a promising, non-cell-based treatment for osteoarthritis while more investigation is required to fully elucidate their mechanism of action.

### 5.2 Anti-apoptotic effects of MSC-EVs

MSC-derived exosomes convey different anti-apoptotic components that have been discovered within different studies. The transportation of small miRs such as 21-5p, 486-5p, and miR-125b have been shown to act as anti-apoptotic elements. In the mice lung ischemia/reperfusion model and the *in vitro* hypoxia/reoxygenation (H/R) model, MSC-derived exosomes inhibited apoptotic pathways and targeted PTEN and PDCD4 carrying miR-21-5p (wei Li et al., 2019). As the most abundant miRNAs in MSC-derived exosomes, Luther et al. described their inhibitory role in myocardium apoptosis by downregulation of pro-apoptotic gene products such as FasL, Peli1, PTEN, and PDCD4 (Luther et al., 2018). Wang et al. discovered the higher expression of exosomal miR‐21 in endometrium‐derived MSCs (EnMSCs) in comparison to the MSCs from other sources such as adipose tissue or bone marrow. They proposed that the exosome anti-apoptotic mechanism of action in myocardial infarction is mediated by miR-21 to target the PTEN‐Akt survival pathway, which directly affects the Bcl2 expression (Wang et al., 2017b). In another study, these exosomes activated the PI3K/AKT pathway by carrying miR-486-5p and subsequently hindered apoptosis of injured cardiomyocytes (Sun et al., 2019). Zhu et al. successfully stimulated murine BMMSC-derived exosome to express miR125b-5p by preconditioning them under hypoxia. These exosomes significantly reduced cardiomyocyte apoptosis in an MI model by suppressing pro-apoptotic genes such as p53 and BAK1 (Zhu et al., 2018). Although the exact principle of action has not been discovered, MSC-derived EVs illustrated significant anti-apoptotic properties in OA models. (BM)-MSCs derived exosomes prevented murine chondrocyte apoptosis in a dose-dependent manner, but showed less efficiency than MSCs (Cosenza et al., 2017). lncRNA KLF3-AS1, thought to be one of the key components in MSC-exosomes, reduces chondrocytes apoptosis by targeting miR-206 (Liu et al., 2018a; Liu et al., 2018b). On the other hand, Mao designed a study to prove the impact of miR-92a-3p in MSC exosomes in controlling chondrocyte degradation. They suggested that this miR by targeting WNT5A can decelerate OA progression and maintain cartilage stability (Mao et al., 2018). In addition, MSC-exosome-mediated apoptosis gene regulation was found to attenuate apoptosis in chondrocytes by targeting Survivin and Bcl-2 (Zhang et al., 2018a). Qi and his colleagues suggested that after administration of MSC-derived exosomes to IL-1β induced chondrocytes, p38 and ERK phosphorylation were suppressed. -Akt showed increased phosphorylation, leading to mitochondrial-induced apoptosis inhibition through IL-1β (Qi et al., 2019). hIPFP-MSC derived EVs also ameliorated OA severity in IL-1β-induced apoptosis of chondrocytes (Wu et al., 2019). Interestingly, 2D and 3D cultured umbilical cord MSC (U-MSCs)-derived EVs showed anti-apoptotic effects on IL-1β-induced chondrocytes (Yan and Wu, 2019). Despite the need for further investigation of exact underlying mechanisms of anti-apoptosis action in MSC-EVs, it is apparent that they are carrying potential components that can be effective in treatment of apoptosis in disorders such as OA.

### 5.3 Chondroprotective effects of MSC-EVs

Chondrocytes, as metabolically active cells, are primarily responsible for extracellular matrix components synthesis and turnover in the joint while they only occupy 1%–5% of total cartilage tissue (Bhosale and Richardson, 2008). During OA development, pro-inflammatory factors, such as cytokines disturb the cell metabolic activity with anabolic and catabolic effects, leading to imbalances between anabolic (synthesis) and catabolic (degradation) processes, and affecting ECM remodeling (Akkiraju and Nohe, 2015). Similar to MSCs, their EVs are capable of restoring the anabolic/catabolic equilibrium in chondrocytes by downregulating catabolic processes, often upregulated in OA. Functional cartilage is compromised through the overexpression of inflammatory cytokines and matrix metalloproteinases (MMPs). Exosomes and microparticles of Rat TGFβ3-pretreated (BM)-MSCs induced the anabolic markers expression such as COL2B and ACAN genes while eliminated the catabolic MMP-13 expression and iNOS inflammatory factor in OA-like chondrocyte (Cosenza et al., 2017). EVs derived from synovial fluid MSCs also induced anabolic marker expression and decreased the expression of catabolic genes in chondrocytes, causing an improvement in ORSI scores and Col II (Liang et al., 2023).

Delivery of miRNAs and growth factors are other key mechanisms of action described for MSC-EVs which exert their chondroprotective properties. Exosomes from MSCs treated with TGFβ3 in mice carried miR-135b and enhanced chondrocyte proliferation and cartilage repair by downregulating SP1 in secreted EVs. KLF3-AS1, one of the pivotal components in hMSC- derived exosomes, increased chondrocyte survival by increasing their proliferation and migration and mitigating their apoptosis after OA induction with IL-1β. In this study, EVs from knock-down KLF3-AS1 MSCs failed to reduce inflammatory responses as opposed to control exosomes (Liu et al., 2018a). MiR-145 and miR-214 in EVs from hASCs cultured with xeno-free medium protected IL-1a-induced chondrocytes (Palamà et al., 2023). EVs secreted from infrapatellar fat pad MSCs (hIPFP-MSC) also improved human IL-1β-induced chondrocyte proliferation through enhancing Col II by prompting mTOR inhibition and autophagy presentation. These EVs target the mTOR mRNA 3′UTR region by delivering miR-100-5p into chondrocytes, leading to lower levels of mTOR and subsequently enhancement of chondrocyte autophagy and repression of catabolism in chondrocytes treated with IL-1β (Wu et al., 2019). Also, hBMSC-derived EVs co-cultured with OA human chondrocytes inhibited pro-inflammatory mediators and COX-2. This resulted in an improvement in expression of WNT7A and Sox-9, while reducing the levels of RUNX2, ALP, and COL10A1 genes that induce hypertrophic differentiation (Vonk et al., 2018). 3D printed (BM)-MSC-exosomes led to downregulation of MMP13 and ADAMTS 5. Additionally, the levels of AGG and Col II were increased in OA chondrocytes. These EVs also restored chondrocyte mitochondrial dysfunction and escalated chondrocyte migration (Chen et al., 2019). MSCs derived from human ESC differentiation produced exosomes that have been applied in a co-culture system with rat chondrocytes. These EVs increased the expression of genes that are associated with anti-apoptotic effects and proliferation, which is mediated by the phosphorylation pathways of ERK, AKT, and CD73 (Zhang et al., 2018a). On the other hand, exosomes from human ESC-derived MSCs co-cultured with rat TMJ condylar in a cartilage model of OA demonstrated a dose-dependent suppression in NO and MMP13 production, while GAG production was enhanced (Zhang et al., 2019). These exosomes were also able to increase Col II and inhibit ADAMTS5 levels in OA-induced mouse chondrocytes (Wang et al., 2017a). Exosomes secreted by human iPSCderived MSCs (iMSC) and synovial membrane MSCs (SM-MSC) have also been compared to evaluating their capacity in OA treatment. Although both EVs affect chondrocyte Col II production and ADAMTS5 expression, iMSC-derived EVs exerted a more potent effect (Zhu et al., 2017). MSC manipulation was also able to generate EVs with the capacity to target OA development. For instance, activation of Yes-associated protein (YAP) was induced by miR-140-5p exosome release from (SM)-MSCs. This led to increased SOX9 expression and improved ECM formation with inhibition of RalA leading to enhancement of human chondrocyte proliferation and migration (Tao et al., 2017). Additionally, overexpression of miR-92a-3p in human MSCs led to release of EVs that increased proliferation and the migration rate of chondrocytes, and also reduced expression levels of MMP13 and WNT5A (Mao et al., 2018). Yan *et al.* also studied chondroprotective effects of umbilical MSCs-derived EVs. These EVs enhanced chondrocytes proliferation, migration and matrix synthesis. It was also revealed that EVs derived from 3D-cultured MSCs exhibited more substantial effects on maintaining chondrocytes phenotypic stability. It was suggested that EVs from 3D culture exert their effects through the TGF-β1-dependent Smad2/3 signaling pathway, which is critical in ECM production and protection of chondrocytes from apoptosis (Yan and Wu, 2019).

### 5.4 Immunosuppressive and anti-inflammatory effects

MSCs and their EVs exert immunosuppressive properties, which support their application in degenerative diseases. MSC-EVs influence a complex network of cells to control immune responses and alter the pro-inflammatory to anti-inflammatory condition after administration. In degenerative diseases, the immune system is involved in onset and progression of symptoms, influencing severity and the extent of tissue damage. In particular, during OA development a complex interaction between the innate and adoptive immune systems creates an environment that promotes tissue damage and drives disease progression.

#### 5.4.1 Natural killer cells (NK cells)

NK cells are amongst the primarily affected cells after being exposed to MSC-EVs. These EVs transfer IL-10, TGF-β1 and human leukocyte antigen-G (HLA-G) as anti-inflammatory components to NK cells. MSC-EV administration to GVHD patients decreased TNF-α and IFN-γ secretion from NK cells (Kordelas et al., 2014). EVs released by human umbilical cord MSCs downregulated the renal expression of toll-like receptor-2 (TLR-2) and C-X3-C motif chemokine ligand-1 (CX3CL1) in rat renal ischemia-reperfusion injury model, which inhibited CD3^−^CD161+NK infiltration and regulated NK cells (Zou et al., 2016). In another study, the role of human fetal liver MSC-exosomes was investigated leading to the suggestion that TGF-β carried by exosomes induces downstream TGFβ/Smad2/3 signaling pathways resulting in the restoration of NK cell proliferation, differentiation and cytotoxicity (Fan et al., 2019). During OA development, synovial tissue infiltrated NK cells demonstrated a immunoregulatory-like phenotype with granzyme A production inducing osteoclastogenesis (Jaime et al., 2017; Huss et al., 2010; Santiago et al., 2017). The interaction between MSC-derived EVs and joint NK cells has not been fully studied as yet, but PGE-2 released by MSCs and IDO together have been found to alter cytokine production by NK cells and it is possible that these components are delivered to NK cells as EV cargo (Spaggiari et al., 2008).

#### 5.4.2 Dendritic cells (DCs)

Dendritic cells (DCs), acting as antigen-presenting cells (APCs) are involved in the innate sensing of pathogens and regulation of pro-inflammatory responses and have a complex role in OA development and progression. It has been suggested that the Toll-like receptor family participates in recognition and activation of innate immunity of DCs after pathogen exposure in the OA joint (Herrero-Beaumont et al., 2019). In a mouse model of high-fat diet (HFD)-induced OA, TLR-4 promoted cartilage catabolism, for example (Kalaitzoglou et al., 2019).

TLR-10 genetic polymorphisms have been introduced as one of the susceptible elements in clinical OA patients. Nie et al. demonstrated the DC overexpression of TLR in OA led to a vigorous inflammatory response clarifying the role of DCs in disease development (Nie et al., 2019). MSC-EVs have been shown to inhibit DC maturation and activation with direct effects on their proliferation and apoptosis (Reis et al., 2018). MSC-EVs induced DC immaturity through upregulation of miR-146a and decreased FAS expression (Wu et al., 2017). MSC-derived decreased dendritic cell surface expression of maturation markers such as CD83, CD86, CD40 and MHCII, leading to reduction of IL-6 while increasing IL-10 and TGF-β release has also shown (Shahir et al., 2020). Reis and colleagues also demonstrated decreased maturation and function after exposing dendritic cells to MSC-EVs. MSC-EV treated dendritic cells had lower levels of CCR7, decreased expression of CD83, CD38, and CD80 markers, decreased secretion of the pro-inflammatory cytokines IL-6 and IL-12p70 and increased production of the anti-inflammatory cytokine TGF-β. Among the top miRs found in these EVs, miR-21-5p, miR-142-3p, miR-223-3p and miR-126-3p were known to have effects on DCs maturation and functionality (Reis et al., 2018). Although the exact role of these EVs on DCs in the OA joint has not been directly investigated, it can be proposed that one of the MSC-EV mechanisms of action in the joint can suppress DC maturation and inhibit activation of the innate immune response.

#### 5.4.3 Macrophages and monocytes

In the OA joint, macrophage activation with production of pro-inflammatory cytokines and MMPs induces cartilage damage and osteophyte formation through hemostatic imbalances (Chen et al., 2020b). During disease development, M1/M2 macrophages are associated with a transformation to the M1 pro-inflammatory subtype, leading to an increase in cytokine overproduction of TNF-α, IL-1β and IL-12 and as well as other catabolic and anabolic markers (Gu et al., 2017). OA severity is linked directly to the presence of activated M1 macrophages in patients (Liu et al., 2018c). On the other hand, macrophage polarization to the M2 phenotype might be a possible therapeutic target for cartilage damage. Several studies demonstrated that MSC-EVs might act as inhibitors of macrophage inflammation by suppressing M1 infiltration and activation. On the other hand, M1 to M2 polarization has been regulated, which can facilitate the M2 immunotolerance response (Henao Agudelo et al., 2017; Morrison et al., 2017; Zhao et al., 2018; Singla et al., 2019). Furthermore, EVs can mediate hemostasis associating mitochondria from MSCs to macrophages to boost their bioenergetics during oxidative stresses (Phinney et al., 2015). Pre-treatment of bone marrow-derived MSCs with IL1β enhanced macrophage polarization toward a M2 phenotype in response to the release of miR-146a-containing exosomes MSCs (Song et al., 2017). Cosenza et al. found that exosomes from MSCs resulted in inhibition of macrophage activation with a reduction of TNF-α expression and IL-10 upregulation (Cosenza et al., 2017). Other miRs were found to act as M2 macrophage modulating factors. MiR-135b increased M2 polarization in an OA rat model with MAPK6 downregulation, for example (Wang and Xu, 2021). Human UC-MSC-derived EVs have been shown to carry miR-100-5p, miR-148a-3p, miR-122-5p, miR-let-7a-5p, and miR-486-5p and mediate OA progression and M2 polarization by regulating the PI3K-Akt pathway (Li et al., 2022a). The role of hUC-MSC-derived exosomes in the OA environment were also tested using the THP-1 human monocyte cell line. Results indicated that these EVs reduced pro-inflammatory factors in M0 and M1 macrophages and also induced anti-inflammatory markers in M0, eliminating OA development with increasing the infiltration of CD163^+^ regenerative M2 macrophages and less inflammatory CD86^+^ M1 macrophages during articular cartilage repair (Li et al., 2022b). Zhang *et al.* also found that MSC exosomes can induce M2 macrophages that infiltrate cartilage defects and synovium in OA cartilage, reduce the infiltration of M1 macrophages and downregulate the expression of IL-1β and TNF-α, thereby inhibiting the inflammatory response in OA (Zhang et al., 2018a).

#### 5.4.4 T-Cells

T-cells are responsible for the majority of synovial infiltration in OA patients joints contributing to disease development. Significant alteration in the profile of T-cell subtypes in synovial fluid, synovial membranes and peripheral blood leads to a multi-aspect functional effect on disease progress. Th1 cells are the dominant T cell subtype in the OA joint with TNF-α, IFN-γ, and IL-2 produced by these cells contributing to joint pain and inflammation (Yamada et al., 2011; Dolganiuc et al., 1999; Hussein et al., 2008; Ishii et al., 2002; Sakkas et al., 1998; Miller et al., 2014). Th2 and Th22 seem to have a relatively minor contribution i to OA progression, while Tregs are decreased substantially in synovial fluid of OA patients, indicating a possible role in controlling disease progression (Ponchel et al., 2015). The effect of EVs derived from MSCs on T-cells has been widely examined revealing a variety of outcomes across different studies. For example, studies have described their role on T-cells as an indirect interaction through DCs and macrophages (Du et al., 2018; Zhang et al., 2018b), while other studies suggested that they can directly suppress T-cell proliferation and upregulate Treg proliferation through factors such as IDO, active CD73 protein, TGF-β or different subsets of miRs (Blazquez et al., 2014; Xie et al., 2020). Treg cell populations (CD4^+^CD25^+^Foxp3^+^) tended to increase in MSC-EVs treated groups while parental MSCs had no effects (Cosenza et al., 2018). MSCs pre-stimulated with inflammatory factors such as TGF-β and IFN-γ showed more immunosuppressive properties and promoted Treg differentiation (Zhang et al., 2018c; Kilpinen et al., 2013). These EVs were also observed to act as a Th1/Th2 balance regulator through engagement in the transformation from pro-inflammatory to anti-inflammatory stages by decreasing TNF-α and INF-γ and stimulating IL-10 or IL-4 production (Cosenza et al., 2018; Nojehdehi et al., 2018). They can also reduce T-cell migration and infiltration to the site of lesions (Farinazzo et al., 2018). As for macrophages, these EVs carry miRs to T-cells and by targeting different pathways control the OA environment. In a report published by Ragni et al. (2021), 223 EV-miRs were identified after exposure of ASCs (adipose derived MSC) to OA synovial fluid, highlighting the role of these miRs in T-cell and Treg proliferation and activation. These EVs suppress the maturation of T cells by downregulating TNF-α and IL-1β (Chen et al., 2016; Del Fattore et al., 2015), while upregulation of IL-10 and TGF-β1 from PBMCs, promoted proliferation and immune-suppression capacity of Tregs (Du et al., 2018). On the other hand, after exposure to PBMCs or CD3^+^ cells proliferation is reduced leading to apoptosis in these cells (Chen et al., 2016). MSC-derived EVs promoted human umbilical cord derived-Treg survival by upregulating the expression of LC3(II/I), phosphorylated Jak3 and Stat5 as well as regulation of FOXP3 expression in the Tregs. This resulted in enhanced T-reg function and stability by activating autophagy and Stat5 signaling pathways (Zhang et al., 2022b). In a mouse model of arthritis, hUC-MSC derived EVs significantly inhibited the expression of IL-22 and increased the levels of miR-29a-3p, miR-29b-3p, and miR-29c-3p in CD4^+^ T cells. In particular, after loading these EVs with miR-29-3p, they inhibited the aryl hydrocarbon receptor (AhR), IL-22, IL-22R1, MMP3, and MMP13 expression in T cells. The AhR/IL-22 signaling pathway in synovial tissue plays a role in regulation of arthritis inflammatory responses (Wei et al., 2025). IFN-γ and TGF-β1-licensed MSCderived EVs enhanced Treg proliferation and suppressed T cell proliferation better than non-licensed MSC EVs as well as inhibiting THP-1 macrophage activation (Wang et al., 2025). T cells are affected by PEG2 released from macrophages that are polarized to M2 subtype and produce more Th2 and decrease T cell proliferation modulating OA progression (Zhao et al., 2020). Similarly, in other conditions such as GVHD, the MSC-EVs enhanced Treg production (CD4^+^CD25^+^ T cells or CD4^+^CD25^+^Foxp3^+^ Tregs) through an APC-mediated pathway when CD4^+^ T cells were activated by APC-enriched CD11C^+^ cells, both *in vitro* and *in vivo*. These EVs also reduced symptoms and increased survival (Zhang et al., 2018b).

#### 5.4.5 B-Cells

While in OA, T-cells control cellular immunity, activated B-cells contribute to humoral immunity development. Interestingly, they have shown an antigen-driven activation that leads to antibody production against cartilage (Shiokawa et al., 2001; Mollenhauer et al., 1988). B-cell proliferation was inhibited by MSC-EVs as well as their differentiation and antibody production (Budoni et al., 2013). Moreover, in the mice treated with these EVs, B-cell activation was reduced (Madel et al., 2023). This might be attributed to their suppressive ability in B-cell inflammation. Incubation of MPs and exosomes with B-lymphocytes showed significant inhibition in plasmablast differentiation and reduced the total produced IgG. These EVs had no effects on cytokine production from differentiating plasmablasts (Cosenza et al., 2018). Considering the effect of MSC-derived EVs on the PI3K-AKT signaling pathway and B regulatory (Breg) cells, these EVs inhibit B cells by disrupting BCR-mediated Ca^2+^ mobilization (Adamo et al., 2019).

## 6 MSCs and their EVs translation: navigating expansion, characterisation and administration challenges

The efficacy of EV treatments in several pre-clinical models of different diseases has prompted interest in moving from mesenchymal stem cell therapies to cell-free therapy.

### 6.1 EV isolation and large-scale expansion

Despite the multiplicity of discovered sources of MSCs, primary isolated bone marrow-derived MSCs (BMSCs) and adipose-derived MSCs (AMSCs) are among the most common sources of MSCs for clinical use in OA treatments (Kim and Keating, 2019). While BMSC harvesting from the posterior superior iliac spine requires an invasive procedure, AMSCs are isolated from the homogenized adipose tissue generally harvested from subcutaneous sites and infrapatellar fat pads. Donor age disparately affects the effectiveness of bone marrow cells and MSCs regenerative properties, including their number, maximal lifespan, and differentiation capacity (Mastrolia et al., 2019). Other sources of MSCs like umbilical blood-derived MSCs and stromal vascular cells (SVF) have been tested at the clinical trial level. Based on successful MSCs trials and preclinical MSC-EVs in OA models, it may be that BMSCs and AMSCs are the best sources for EV production. However, the source of MSCs and culture conditions can alter the EV yield and biological activity. For example, MSCs cultured under serum starvation, compared to serum-containing culture conditions, produce different EV subtypes (Kim et al., 2021). With current isolation methods, the separation of non-MSC EVs from MSC-EVs is not possible.

On the other hand, cell count for EV production is a critical factor that is still not standardized for clinical treatments. In addition to cell source and serum, other elements like oxygen levels and cell confluency influence the EV yield and potential. Hypoxia culture conditions promote EV secretion, activating other pathways by transferring components to the targeted tissue (Zhang et al., 2022a; Sicco et al., 2017; Lee et al., 2012; Zhang et al., 2012). Cell confluency and contact inhibition may prime the cells to enter quiescence leading to lower EV production or lower therapeutically potent EV secretion (Steinman et al., 2003; Hayes et al., 2005). For MSC therapy, after cell administration of 2-200 x 10^6^, a statistically significant improvement in clinical outcome was observed (Barry, 2019). However, administration of EV quantities requires more investigation in larger animal models before moving to clinical trials. By translating rodent MSC-derived EV production (2 million MSCs) to a human dose, Alderz et al. suggested 500 million MSCs would be required for sufficient EV collection (Adlerz et al., 2020). Large-scale MSC expansion with sustained conditions for EV production raises the potential of 3D culture systems. Fully automated bioreactors ease the monitoring and control of process parameters but still alter EV yield and characteristics (Hahm et al., 2021). Interestingly, MSCs cultured in bioreactors enabled higher EV production (Almeria et al., 2024). Also, 3D-cultured iMSC-derived EVs were comparable to 2D-cultured-MSC EVs in terms of protein and lipid content, size and phenotype (Garcia et al., 2024). MSC-EVs also exhibited superior chondroprotective effects both *in vitro* and *in vivo* compared to EVs collected from 2D-cultured MSCs (Yan and Wu, 2019). If EV properties remain untouched or improve in 3D systems, bioreactors can scale up EV manufacturing and expedite the production at a lower cost (Zhang et al., 2022a; Sicco et al., 2017; Lee et al., 2012; Zhang et al., 2012). Cell confluency and contact inhibition in fully confluent conditions may promote cell quiescence and lead to lower EV production or lower therapeutically potent EV secretion (Steinman et al., 2003; Hayes et al., 2005). For MSCs therapy, after 2-200 × 10^6^ cell administration, a statistically significant improvement in clinical outcome was observed (Barry, 2019). However, EV quantities require more investigation in larger animal models before moving to clinical trials. By translating rodent MSC-derived EV production (2 million MSCs) to a human dose, Alderz et al. suggested 500 million MSCs would be required for sufficient EV collection (Adlerz et al., 2020). Large-scale MSC expansion with the sustained condition for EV production raises the potential of 3D culture systems. Fully automated bioreactors ease the monitoring and control of process parameters but still alter EV yield and characteristics (Hahm et al., 2021). Interestingly, MSCs cultured in bioreactors enabled higher EV production (Almeria et al., 2024). Also, 3D-cultured iMSC-derived EVs were comparable to 2D-cultured-MSC EVs in terms of protein and lipid content, size, and phenotype (Garcia et al., 2024). MSC-EVs also exhibited superior chondroprotective effects both *in vitro* and *in vivo* compared to EVs collected from 2D-cultured MSCs (Yan and Wu, 2019). If EV properties remain untouched or improved in 3D systems, bioreactors can scale up EV manufacturing and expedite the production with lower costs.

However, EV isolation remains a significant limitation. Lab-scaled commercial purification methods with high EV purity are more accessible for research with ultracentrifugation (UC) and tangential flow filtration (TFF) used in EV bulk production. However, low EV recovery and high impurity levels after UC have made technology attractive for manufacturers. TFF is suitable for enriching EVs from large volumes in a time-effective manner but requires expensive equipment. On the other side, fast protein liquid chromatography (FPLC) and Size Exclusion Chromatography (SEC) enriched EVs with higher purity but lower recovery and yield but generated EVs were shown to have a higher therapeutic potential (Malvicini et al., 2024).

### 6.2 EV characterization

Minimal International Society for Cellular Therapy (ISCT) criteria have been established to identify MSCs as a GMP-grade cell product. However, MSCs are a heterogeneous population of plastic-adherent cells isolated from different sources. They must express defined cell surface markers (CD73, CD90, CD105) and display a negative profile for other markers (CD45, CD34, CD14, and HLA-DR). Also, the differentiation potential of MSCs into osteoblasts, adipocytes and chondroblasts under optimal *in vitro* culture conditions should be performed to verify conformity with these criteria (Horwitz et al., 2005; Dominici et al., 2006).

The International Society for EVs (ISEV) also proposed Minimal Information for Studies of EVs (“MISEV”) to describe EV specific characteristics prior to applying them as therapeutics. Although EV characteristics may vary depending on the cell source and culture conditions, some of the main characteristics remain consistent amongst all different types. First, specific EV markers including tetraspanins are considered as one of the common features observed in all EV subtypes. In most pre-clinical studies, small EVs or exosomes have shown similar potential to their paternal MSCs. However, to ensure EV quality, their size distribution using advanced technologies like the nano tracking assay (NTA), TEM or analysis by a Zetasizer needs to be evaluated. While physical characteristics of EVs receive more attention, their therapeutic potential is not reflected by these characteristics. As mentioned earlier, these EVs act in the disease environment through different compounds, but the exact underlying mechanism of action still needs deeper investigation. It is critical to define a uniform and informative immune potency assay reflecting the EVs immune suppression effect in joints after administration.

While physical and biochemical properties of EVs, including particle size, morphology, and canonical surface markers such as CD9, CD63, and CD8 are now relatively well established through MISEV-based recommendations, the field still lacks standardised functional potency assays that are uniquely designed for each condition and predict their *in vivo* therapeutic efficacy. This gap is one of the major barriers to clinical translation. For MSC products, ISCT and related consensus efforts have emphasized disease-specific potency assays that reflect the intended mechanism of action rather than generic identity markers, and the same principle applies to MSC-EVs. For OA-targeted MSC-EVs, a potency assay should ideally measure a functional endpoint relevant to disease biology, such as suppression of IL-1β-induced catabolic responses in chondrocytes or inhibition of inflammatory macrophage activation, rather than relying only on particle count or marker expression. On the other side, batch-release criteria for clinical-grade EV products should include multiple layers of quality control. Standardised EV identity and purity tests should confirm EV-associated markers and appropriate vesicle morphology, assess protein contaminants, residual nucleic acids, and other process-related impurities. Similarly, potency assays should demonstrate a reproducible biological activity linked to the intended indication, such as immunomodulatory or chondroprotective effects. The need for validated potency assays is particularly urgent because many preclinical studies still rely on heterogeneous EV preparations described mainly by size and concentration, which makes cross-study comparison and dose extrapolation difficult.

### 6.3 Comparative assessment of MSC tissue sources for EV production

The tissue source of MSCs can influence the yield, composition, and therapeutic potential of the produced EVs. Similar to MSCs characteristics which are influenced by their source, EVs from the most commonly used sources of MSC, bone marrow and adipose tissue, carry distinct advantages and limitations for clinical application and manufacturing. BMSCs have a long preclinical and clinical research history due to their distinctive properties, and similarly, their EVs have demonstrated strong and robust chondroprotective and immunomodulatory effects in OA models (Warmink et al., 2023; Cosenza et al., 2017). However, one of the main limitations of BMSCs is that bone marrow harvest is an invasive procedure, donor age significantly reduces MSC yield and potency (Heyman et al., 2025), and the resulting EV quantities may be insufficient for clinical dosing without extensive expansion. AMSCs, isolated from subcutaneous fat or infrapatellar fat pads, are more readily accessible in larger quantities and their EVs have shown promising anti-inflammatory and chondroprotective properties (Palamà et al., 2023). Other factors such as donor metabolic status and obesity can affect AMSC biology and consequently EV cargo composition, introducing variability to the final product (Huang et al., 2023).

Perinatal tissue sources including umbilical cord (Wharton’s jelly), umbilical cord blood, and placenta represent a particularly attractive alternative for clinical EV production because they are safe and non-invasive sources that are easily available with higher proliferative capacity and lower immunogenicity and the removal of donor age as a factor. Wharton’s jelly MSCs in particular have demonstrated potent immunosuppressive properties and their EVs carry chondroprotective miRNAs such as let-7e-5p (Chen et al., 2022). Umbilical cord MSC-derived EVs have shown effective M2 macrophage polarisation and anti-inflammatory effects in OA models (Zhou et al., 2022).

MSCs derived from the joint synovial membranealso have inherent chondrogenic potential and their EVs have shown efficacy in OA models (Tao et al., 2017; Liang et al., 2023). However, their harvest requires arthroscopic procedures with limitations of production scalability.

In summary, while no single source has been definitively established as superior, perinatal sources, particularly Wharton’s jelly and umbilical cord blood may be the best candidates for clinical EV manufacturing due to the combination of availability, scalability, donor-independent consistency and demonstrated therapeutic activity. Adipose tissue and bone marrow have been used in EVs studies more critically and their properties have been more fully investigated. Future comparative studies directly evaluating EV potency across sources under standardised conditions are urgently needed to guide source selection for clinical translation.

## 7 Conclusion

This review gives a positive answer to the central question in the field: Do EVs have superior therapeutic potential compared to MSCs in OA treatment? While MSCs have already demonstrated promising therapeutic effects in preclinical and early clinical stages for safety and efficacy, their translation to the clinic remains challenging due to high manufacturing variability, immunogenicity concerns, risk of unwanted differentiation and inconsistent and negative clinical trial outcomes. EVs directly address many of these limitations, making large scale production feasible as they are rigorously characterized and available off-the-shelf with less concern for immunogenicity or tumorgenicity potential, making allogeneic sources more suitable. Like MSCS, EVs show properties such as chondroprotection, immunomodulation, anti-inflammatory effects, and subchondral bone remodeling while potentially offering more targeted and controllable delivery of their cargo. In addition, EVs can be modified and engineered to carry the more potent therapeutic components.

However, it must be acknowledged that the role of EVs in the OA environment is still not fully clear. Similar to MSCs, studies in the field are suffering from the same limitations such as effects of donor characteristics, tissue source, culture conditions, and isolation methods. The field currently lacks standardisedpotency assays, and the optimal dose, route, and frequency of administration remain undefined. The very limited number of clinical trials (nine registered as of this review) reflects how early the field remains in its translational journey.

Although the EV field is rapidly expanding, and cell-free therapy holds great promise as an alternative approach for many diseases, there are still unknown aspects that need to be addressed. As stated in this review, MSCs are the most studied cell source to understand EV roles in OA and other disease treatments.

Despite the complexities of OA, MSCs are known as the forefront of advanced OA therapies. However, MSC clinical trials have not shown a strong track record of successful disease control in the context of both symptom relief and slowing disease progression. More focus on MSCs mechanisms of action in OA might highlight the prominent role of different secreted factors alongside their EVs. Besides the cell-cell interaction in the joint environment, MSCs undergo apoptosis shortly after administration by the host immune system. It means that before and after exposure to disease conditions, MSCs might exhibit different properties, which can be mirrored in their EV characteristics.

On the other hand, MSC clearance in the joint may indicate that after exposure to various factors, cells undergo apoptosis, leading to apoptotic body production. It could be suggested that this phenomenon may underpin EV production, which contributes to their therapeutic effects in OA. However, current studies are limited to specific subtypes of EVs at smaller size, while bigger EVs such as apoptotic bodies may be more effective in the OA environment. Cosenza et al. have shown that both MSC-derived MPs and exosomes have a substantial impact on OA development (Cosenza et al., 2017). Variations in isolation methods and MSC sources in outlined studies could impact this decision (Álvarez-Viejo, 2020). Because of the distinctive structure of EVs, there is no available technique to purify them without other contaminants; in the majority of methods used, isolated samples are contaminated with other secreted factors. However, when comparing the effects of EVs, the cell secretome and the parental cells, it is seen that they have similar outcomes after administration, although they may operate through different pathways. In light of this, MSC-derived EVs may represent a potent candidate for OA therapy, but before shifting from MSCs to EVs, further research is needed to fully understand the composition and function of isolated EVs, their exact mechanisms of action, optimal dosage and route of administration.

To achieve clinical translation, research must prioritise the following directions (Zhang et al., 2008): establishment of standardized, disease-specific potency assays that predict *in vivo* efficacy (Roubille et al., 2013); more concise comparative studies of EVs from different tissue sources under uniform conditions (Mautner et al., 2023); development of scalable, GMP-compliant manufacturing process (Pers et al., 2025); systematic investigation of EV biodistribution and cellular targets within the OA joint (Ankrum et al., 2014); well-designed, adequately powered clinical trials with standardised patient stratification, dosing protocols, and outcome measures. Only through this rigorous translational pathway can the considerable promise of MSC-EV therapy for OA be realised.
